# Psychedelic-assisted Therapy Training: Firsthand Experience of Non-Ordinary States of Consciousness in the Development of Competence

**DOI:** 10.1192/j.eurpsy.2024.1660

**Published:** 2024-08-27

**Authors:** S. Dames, C. Watler, P. Kryskow, P. Allard, M. Gagnon, V. Tsang

**Affiliations:** ^1^ VIU; ^2^Roots to Thrive, Nanaimo; ^3^UBC, Vancouver, Canada

## Abstract

**Introduction:**

This review explores the benefits of incorporating personal experience(s) with non-ordinary states of consciousness as a core component of Psychedelic-Assisted Therapy (PaT) training.

The program incorporates an optional experiential training component. We collaborate with professionals affiliated with a Canadian non-profit organization specializing in PaT experiential training. As do other stakeholders in this field– including program developers, educators, and researchers–we navigate a rapidly evolving and often ambiguous landscape, where infrastructure and regulations are lagging scientific data and best practices. Given the potential for differing perspectives, the authors acknowledge that their personal experiences could be a potential source of bias, influencing objectivity.

**Objectives:**

Conversely, these lived experiences could be seen as valuable contributions, enriching perspectives on the role of experiential training. In that context, our intention is to provide a comprehensive review, presenting arguments both in favour of and against the integration of experiential training in PaT.

**Methods:**

There is an urgent need for establishing legal training and practice options, bridging the underground with best practices, with all practitioners operating within a regulated and ethically accountable framework. Such a proactive strategy would mitigate the risks associated with unregulated training in a field with relatively few guidelines on how to develop competency.

**Results:**

An in-training PaT experience supports personal comfort, self-assuredness, and confidence supporting others in non-ordinary states of consciousness, with contemporary researchers/experts highlighting the specific challenges among therapists who lack lived experience. These might include holding unrealistic expectations, being unaware of the impacts of set and setting, and misunderstanding

**Conclusions:**

Whether or not therapists engage in experiential training - serving a dual in one’s own healing process, it is imperative that they maintain their own wellness practices. This proactive/primary prevention strategy would improve well-being and resilience, reducing secondary mental health consequences for patients and providers. Cultivating a culture of self-care within the mental health field should be an overarching priority for training programs and professional organizations, without which we are left with broken people in support of broken people. Current rates of burnout, absenteeism and early retirement suggest that we are already on that trajectory and should serve as a call to action.

**Disclosure of Interest:**

None Declared

